# Roles of the Tol/Pal System in Bacterial Pathogenesis and Its Application to Antibacterial Therapy

**DOI:** 10.3390/vaccines10030422

**Published:** 2022-03-10

**Authors:** Hidetada Hirakawa, Kazutomo Suzue, Haruyoshi Tomita

**Affiliations:** 1 Department of Bacteriology, Graduate School of Medicine, Gunma University, Maebashi 371-8511, Gunma, Japan; tomitaha@gunma-u.ac.jp; 2 Department of Infectious Diseases and Host Defense, Graduate School of Medicine, Gunma University, Maebashi 371-8511, Gunma, Japan; 3 Laboratory of Bacterial Drug Resistance, Graduate School of Medicine, Gunma University, Maebashi 371-8511, Gunma, Japan

**Keywords:** Gram-negative bacteria, virulence, drug resistance, antimicrobial chemotherapy, vaccine, outer membrane protein, bacterial pathogenesis

## Abstract

The Tol/Pal system (also written as “The Tol-Pal system”) is a set of protein complexes produced by most Gram-negative bacteria. It comprises the inner membrane-associated and the outer membrane-anchored subunits composed of the TolA, TolQ, and TolR proteins and the TolB and Pal proteins, respectively. Although the Tol/Pal system was first defined as bacterial proteins involved in colicin uptake of *Escherichia coli*, its global roles have been characterized in several studies as mentioned in this article. Pathogenesis of many Gram-negative pathogens is sustained by the Tol/Pal system. It is also essential for cell growth and fitness in some pathogens. Therefore, the Tol/Pal system is proposed as a potential target for antimicrobial chemotherapy. Although the *tol*/*pal* mutants are low in virulence, they still have the ability to stimulate the immune system. The Pal protein is highly immunogenic and induces both adaptive and innate immune responses. Therefore, the *tol*/*pal* mutant strains and Pal proteins also have potential vaccine properties. For these reasons, the Tol/Pal system represents a promising research target in the development of antibacterial therapeutic strategies for refractory infections caused by multi-drug-resistant (MDR), Gram-negative pathogens. In this paper, we summarize studies on the Tol/Pal system associated with bacterial pathogenesis and vaccine development.

## 1. Introduction

Antimicrobial agents are commonly used for the prevention and treatment of bacterial infections; however, an increasing number of bacteria have acquired resistance to conventional drugs. On account of this, there is an urgent need to develop alternative chemotherapy options, such as new drugs and vaccines.

Gram-negative bacteria ubiquitously have a lipid bilayer named the outer membrane, outside the cell wall and cytoplasmic inner membrane. The outer membrane is a permeability barrier that limits the entry of drugs into the cells [1]. Due to this, most Gram-negative bacteria are innately tolerant to some classes of antimicrobial agents [2,3]. They are hard to treat when additional resistance is acquired. Many classes of antimicrobial agents have been used in medical and industrial applications to prevent and treat infectious diseases caused by these pathogens, and their widespread application has resulted in the development of drug-resistant bacteria.

**Figure 1:**
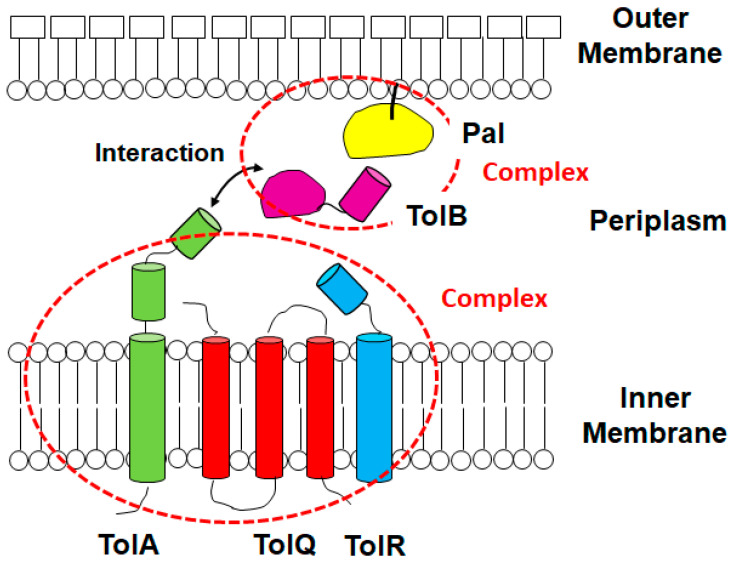
Structure representation of the Tol/Pal protein complexes. TolA, TolQ and TolR proteins form a complex in the inner membrane while the periplasmic TolB protein is associated with the outer membrane-associated Pal protein at the C-terminal site. The N-terminal domain in the TolB protein interacts with the C-terminal domain in the TolA protein.

The Tol/Pal system is a set of protein complexes. It is composed of the TolA, TolB, TolQ, TolR, and Pal proteins, and they form two protein complexes that traverse the outer membrane, periplasmic space and inner membrane [4]. Although the *tol*/*pal* genes were originally identified in *Escherichia coli*, these orthologous genes are found in many Gram-negative bacteria [5]. The *tol*/*pal* genes are transcribed in two operons (*ybgC*-*tolQ*-*tolR*-*tolA* and *tolB*-*pal*-*cpoB*), while a single large (*ybgC*-*tolQ*-*tolR*-*tolA*-*tolB*-*pal*-*cpoB*) transcript is also observed [6,7]. Expression of *tol*/*pal* genes is regulated by RcsC, a sensor kinase of the two-component regulatory system, in *E. coli*, iron in *Pseudomonas aeruginosa* and CRP (cyclic AMP receptor protein) in *Klebsiella pneumoniae* [8,9,10]. The TolQ, TolR and TolA proteins form a complex in the inner membrane while the periplasmic TolB protein interacts with the Pal protein anchored in the outer membrane [11,12] (Figure 1). The TolQ-TolR-TolA inner membrane complex has structural similarities to the TonB-ExbB-ExbD iron/siderophore uptake system and the MotA-MotB flagellar motor complex [13,14]. Some in vitro studies show that TolB binds to the C-terminal domain of the TolA protein [15,16,17]. TolB can also interact with several proteins localized in the outer membrane, including OmpA, OmpF, and Lpp [18]. The function of the Tol/Pal system is disrupted when any of the *tolA*, *tolB*, *tolQ*, *tolR* and *pal* genes are mutated. The *ybgC* and *cpoB* (formally named *ybgF*) genes encode cytoplasmic lipid thioesterase and the periplasmic regulator of peptidoglycan peptide crosslinking, respectively [19,20]. Neither YbgC nor CpoB participates in the Tol/Pal system because these mutations do not yield the same phenotypes as *tol/pal* mutants. Mutations in the *tol*/*pal* genes have a pleiotropic effect [21]. The *tol*/*pal* genes were originally characterized as genes associated with tolerance to colicins that are bacteriocins produced by *E. coli* [22]. The *tol*/*pal* mutants were shown to be resistant to colicins with a low ability of colicin uptake, resulting in the cells escaping death. The following studies showed that mutations in *tol*/*pal* genes disturb the outer membrane integrity, induce the formation of mucoid colonies with increased production of colonic acid, and decrease the translocation of filamentous bacteriophages into the cytoplasm [4,8,23,24,25,26]. Remarkably, the disturbance of the outer membrane barrier leads to increased susceptibility to some antimicrobial agents, including colistin, β-lactams, vancomycin and novobiocin [27,28,29,30]. The *tol*/*pal* genes are also involved in the process of bacterial cell division and morphology, and inactivation of these genes induces filamentation of cells in several bacteria, such as *E. coli*, *Pseudomonas putida*, *Vibrio cholerae* [31,32,33].

There is increasing evidence that the Tol/Pal system is also required for virulence in many species of Gram-negative bacteria, and cell growth in some species. Therefore, the Tol/Pal system is a potential drug target while the *tol*/*pal* mutants may serve as potential live attenuated vaccines as they can significantly induce immune responses. Additionally, several studies showed that the Pal protein behaves as one of the bacterial surface antigens and induces an immune response although no clinical evidence has been provided, yet. In the following sections, we describe a series of studies focusing on the roles of the Tol/Pal system in bacterial pathogenesis. We also discuss the potential of Tol/Pal proteins in drug and vaccine development.

## 2. Roles of the Tol/Pal System in Pathogenesis

The involvement of the Tol/Pal system in bacterial pathogenesis has been described in studies that analyzed *tol*/*pal* mutants (summarized in Table 1). Alleviated bacterial burden in hosts infected with the mutants, reduced fitness and/or defective production of certain bacterial proteins required for their virulence, such as toxins and flagellar proteins, were observed. In some pathogens, the *tol*/*pal* genes are essential for growth and/or survival, i.e., *tol*/*pal* mutants cannot replicate and/or survive in the host.

**Table 1:** Roles of the Tol/Pal system in Gram-negative pathogens.

Bacterial Species	Pathogenesis	References
Enterohemorrhagic *Escherichia coli* (EHEC)	Secretion of the T3SS effector proteins, A/E lesion formation, Flagellar synthesis	[34]
*Citrobacter rodentium*	Lethality and Enteritis in mouse	[34]
Uropathogenic *Escherichia coli* (UPEC)	Flagellar synthesis, Bacterial colonization within bladder epithelial cells and in the urinary tract of mice	[35]
*Escherichia coli*	Development of sepsis	[36]
*Salmonella enterica* (Typhimurium)	Bacterial survival in macrophage and mouse, Innate tolerance to bile acid and serum	[37,38,39,40]
*Salmonella enterica* (Choleraesuis)	Lethality in mice, innate tolerance to deoxycholate and vancomycin	[41]
*Shigella flexneri*	Bacterial invasion and growth in epithelial cells, Innate tolerance to antibiotics, bile acid and human complement	[42]
*Pseudomonas aeruginosa*	Bacterial growth	[43,44,45]
*Burkholderia cenocepacia*	Bacterial adhesion to lung cells, Lethality in *G. mellonella*	[46]
*Klebsiella pneumoniae*	Innate tolerance to serum and phagocytosis, Lethality in mice	[47,48]
*Haemophilus ducreyi*	Papules formation, Bacterial survival	[49,50]
*Vibrio cholerae*	Uptake of the CTXphi phase, Bacterial growth at high temperature	[33]
*Edwardsiella ictaluri*	Mortality in catfish	[51]
*Dickeya dadantii* (*Erwinia chrysanthemi*)	Activity of pectinolytic enzyme, Motility, Tissue injury on plant leaf	[52]

### 2.1. Escherichia coli Pathogenic Subgroups

Although most *E. coli* strains settle in the intestine as members of normal intestinal flora, there are a few subtypes that cause severe infectious diseases. Enterohaemorrhagic *E. coli* (EHEC) is a well-known pathogenic subtype. EHEC produces effector proteins that are secreted via transporter machinery, termed “the type III secretion system (T3SS)” [53,54]. The effector proteins enable the bacteria to tightly attach to and injure the intestinal cells (This pathogenesis is commonly named “Attaching and effacing [A/E] lesion”) [55]. Therefore, the effector proteins and their transport machinery are required for EHEC pathogenesis. Deletion of *tolB* was shown to decrease the secretion of the effector proteins and the ability of EHEC to induce A/E lesions [34]. The *tolB* mutant of *Citrobacter rodentium*, the alternative model pathogen to evaluate the T3SS-associated virulence in mice, was avirulent because the mutant caused neither lethality nor enteritis symptoms in mice [34]. Thus, TolB is a potential target for the development of drugs to treat infection caused by EHEC.

Uropathogenic *E. coli* (UPEC) is another pathogenic subtype, and it causes urinary tract infections (UTIs). This bacterium is the most common causative agent for both uncomplicated and complicated UTIs [56]. When UPEC enters the urinary tract, the bacteria initially invade bladder epithelial cells, where they form multicellular microbial colonies [57]. The flagella of UPEC are required for bacterial colonization within bladder epithelial cells [58,59]. The *tol/pal* mutants produce defective flagella, resulting in the mutants forming colonies within the bladder epithelial cells, to a lesser extent [35]. Flagella also contribute to bacterial ascent from the bladder and entry into the kidneys [60,61]. Inoculation of *tolB* deletion mutants into the urinary tract of mice showed a relatively lower bacterial burden and colonization in the kidneys [35]. *E. coli* can cause bloodstream infections as a complication of UTIs and lead to sepsis. Pal was shown to be required for optimal virulence in septic mice because mutant *E. coli* strain with reduced levels of Pal or truncated Pal had lower abilities than the wild-type, to survive in blood and kill the mice [36]. Thus, the Tol/Pal system also contributes to the extraintestinal pathogenesis of *E. coli*.

### 2.2. Salmonella enterica

*Salmonella enterica* represents a major family of intestinal bacterial pathogens. *S. enterica* serovar Typhimurium (S. Typhimurium) is widely used as the alternative model pathogen for *S.* Typhi that causes human typhoid fever [62]. This bacterium causes similar typhoid disease in mice. It also causes diarrhea in humans. *S. enterica* can survive within macrophages by inhibiting the phagolysosome formation and thereby leads to systemic infection [63]. The *tol/pal* mutants were shown to have a low ability to survive in mouse macrophages and systemically infected mice [37,38]. The Tol/Pal system is also responsible for outer membrane homeostasis and resistance to bile acids and serum that aid bacteria to establish infections, mutations of the *tol*/*pal* genes alter the levels of glycerophospholipid (GPL) and lipopolysaccharide (LPS) within the outer membrane, and increase susceptibility to bile acids and serum, respectively [37,38,39,40]. *S. enterica* serovar Choleraesuis (S. Choleraesuis) is a non-typhoidal Salmonella and it causes diseases in pigs. The *tolA*, *tolB* and *tolR* mutants of this bacterium exhibited high sensitivities to deoxycholate and vancomycin with defective envelope integrity and motility [41]. These mutants also exhibited higher LD_50_ values than the wild-type strain in mice [41].

### 2.3. Shigella flexneri

*Shigella flexneri* is a *Shigella* species that causes human shigellosis [64]. This bacterium invades intestinal epithelial cells, causing severe inflammation and death of the cells. During infection, the bacteria can multiply intracellularly and spread to neighboring epithelial cells [65]. However, the *tolR* mutant exhibited low rates of invasiveness and growth within the cultured human epithelial cells and attenuated virulence, as defined by body weight loss, in mice [42]. This mutant was also shown to be highly sensitive to some chemical compounds, including antibiotics, bile salts, and human complement, affecting its fitness and survival in the host. 

### 2.4. Pseudomonas aeruginosa

*Pseudomonas aeruginosa* is an opportunistic pathogen and is often isolated as the causative agent of nosocomial infections. Since this bacterium is innately resistant to some classes of antimicrobial agents, chemotherapy options are limited [66]. In addition, most *P. aeruginosa* strains have a high ability to form a biofilm [67,68]. The important properties of this biofilm are extreme resistance to many conventional antimicrobial agents and host immune systems [69,70]. Therefore, biofilm formation enables bacteria to survive within the host, progressing the infection to an untreatable chronic state. One study showed that TolB is one of the most abundant periplasmic proteins in *P. aeruginosa* [43]. In a transcriptome analysis, it was shown that expression of the *P. aeruginosa tol*/*pal* genes was increased in the biofilm [44]. On the other hand, no *P. aeruginosa* strains with either of *tol*/*pal* genes inactivated, was obtained in conventional site-directed and random transposon mutagenesis methods [71,72,73,74]. Lo Sciuto et al. alternatively constructed a conditional *tolB* mutant by replacing the chromosomal *tolB* with an exogenous copy of the *tolB* gene under the control of an arabinose-dependent promoter [45]. The mutant was unable to grow when arabinose was absent in a medium, which indicated that TolB was indeed essential for the growth of *P. aeruginosa* and might be a good target for the development of drugs to combat *P. aeruginosa* infection.

### 2.5. Burkholderia cenocepacia

*Burkholderia cenocepacia* is a member of the *Burkholderia cepacia* complex of Gram-negative bacteria. This bacterium is a causative agent of chronic respiratory infections and is often isolated from people with cystic fibrosis (CF) [75]. Deletion of the *pal* gene was shown to reduce the ability of *B. cenocepacia* to attach to lung epithelial cells derived from a CF patient [46]. The *pal* mutant also exhibited a lower lethality in *Galleria mellonella* than the wild-type parent.

### 2.6. Klebsiella pneumoniae

Although *Klebsiella pneumoniae* is a normal bacterial member that settles in the mouth, skin, and intestines, it can cause infections in the lungs and the urinary tract, resulting in pneumonia and bacteremia, respectively [76]. The *pal* mutant of *K. pneumoniae* was shown to be more sensitive to serum and macrophages and lesser virulent in mice with septicemia, compared to the wild-type parent [47]. One study demonstrated that the Pal protein is released during bloodstream infections [48]. Thus, Pal of *K. pneumoniae* is closely associated with the development of bloodstream infections caused by the bacteria.

### 2.7. Haemophilus ducreyi

*Haemophilus ducreyi* is the etiologic agent of genital ulcer disease known as chancroid. The virulence of this bacterium can be experimentally estimated by measuring the area of papules, formed on the arms of healthy humans inoculated with the bacterial strain [77]. Pal of *H. ducreyi* was found as an outer membrane protein, with a significant homology to the Pal protein of *E. coli* [78]. This protein was shown to be abundantly expressed in the human model of experimental infection. The *pal* mutant *H. ducreyi* formed papule lesions to a lesser extent than the wild-type [49]. Thus, the expression of Pal contributes to the ability of *H. ducreyi* to develop the pustular disease. Moreover, Pal of *H. ducreyi* was shown to be important for its survival because treatment with anti-Pal sera significantly decreased the bacterial colony-forming units [50].

### 2.8. Vibrio cholerae

Cholera is one of the most common infectious diseases in the world, and it is caused by *Vibrio cholerae* [79,80]. Cholera toxin is the most important causative factor of the disease. CTXphi is a temperate bacteriophage carrying DNA code for cholera toxin, and it infects *V. cholerae* [81]. When its phage DNA is inserted into the bacterial chromosome, the lysogenized strains produce a toxin that causes severe diarrheal disease. The pathogenesis of *V. cholera* is closely associated with the ability to acquire the CTXphi phage. Heilpern et al. found one set of *tolQRAB* orthologues in a *V. cholerae* strain with significant similarity to a corresponding sequence in *E. coli* [33]. The *tolQ*, *tolR*, *tolA* and *tolB* mutants of *V. cholerae* had growth defect phenotypes when cultured at 42 °C. The *tolA*, *tolQ* and *tolR*genes of *V. cholera* were shown to be necessary for the uptake of CTXphi. In contrast, *tolB* was unlikely to play an essential role in this process since the *tolB* mutant could be infected with CTXphi in a similar efficiency as that of the parent strain. This implies a biological function of TolB that does not depend on other Tol/Pal members although its molecular mechanism remains unknown.

### 2.9. Edwardsiella ictaluri

*Edwardsiella ictaluri* is a pathogen for fish, including many catfish species. It causes acute septicemia or chronic meningoencephalitis, called the enteric septicaemia of catfish (ESC) disease [82]. A set of homologous ORFs to *tol*/*pal* genes of *E. coli* was found in the chromosomal DNA of *E. ictaluri*. Abdelhamed et al., demonstrated that the *tolQ* and *tolR* genes contribute to the virulence of this bacterium [51]. Mortalities in catfish experimentally infected with the *tolQ* and *tolR* mutants were significantly lower than in those infected with the parent strain.

### 2.10. Dickeya dadantii (Formerly Named Erwinia chrysanthemi)

*Dickeya dadantii* is a phytopathogenic bacterium. It causes diseases, including necrosis, blight, and soft rot, in many plant species. The bacteria degrade the plant cell wall with the use of pectinolytic enzymes and lead to disruption of the parenchymatous tissues [83]. In addition, oligosaccharides, the degradation products from pectin, are used as growth substrates by the bacteria [84]. The *tol/pal* genes were shown to sustain the activity of pectinolytic enzymes and virulence in *D. dadantii* [52]. However, the activity of pectate lyase produced by the *tolA*, *tolB*, *tolQ* and *pal* mutants was not as virulent as that of the parent *D. dadantii*. The mutants induced tissue injury on plant leaves to a lower degree, compared to the parent strain. Motility also contributes to optimal virulence of *D. dadantii* [85]; these mutants were shown to be less motile than the parent strain [52].

## 3. Immunogenicity of the *tol*/*pal* Mutants and Outer Membrane Vesicles (OMVs)

Interestingly, though *tol*/*pal* mutants of some pathogens are avirulent or exhibit low virulence, they can still induce immune responses. Thus, the *tol*/*pal* mutant strains are potential attenuated live vaccines. The *tolB* mutant of *C. rodentium*, alternatively used to evaluate EHEC virulence in vivo, has an ability to induce the production of immunoglobulin G (IgG) and several cytokines, including IFN-γ and IL-17 and confer protection against the lethal parental wild-type strain [34]. Inoculation with the less-virulent *tolA* mutant strain protects mice against subsequent S. Typhimurium infection [86]. In comparison with naïve control mice, the mice immunized with the *tolA* mutant exhibited significantly lower bacterial burden when infected with the wild-type subsequently. All the mice immunized with the *K. pneumoniae pal* mutant could thus survive after infection with the highly virulent wild-type strain [47]. The *tolQ* and *tolR* mutants of *E. ictaluri* were also shown to provide resistance to infection with the highly virulent wild-type *E. ictaluri* [51].

Many Gram-negative bacteria release outer membrane vesicles (OMVs), which contain various immunogenic bacterial surface components, such as outer membrane proteins, phospholipids, and LPS [87,88]. The *tol*/*pal* mutants in some pathogens, including Salmonella, Shigella, *Helicobacter pylori*, and *Buttiauxella agrestis*, were shown to release a large amount of OMVs with an ability to induce immune responses [41,42,89,90,91,92]. Administration of OMVs from S. Choleraesuis wild-type, and the *tolA*, *tolB*, and *tolR* mutants conferred immunity in mice [41]. Compared to OMVs from the wild-type, mice immunized with OMVs from the *tolA* and *tolR* mutants exhibited a more prolonged survival after a lethal challenge. Interestingly, immunization with OMVs from the *tolB* mutant conferred 40% protection to mice. Thus, OMVs from the *tolB* mutant conferred stronger immunity in mice compared to OMVs from the *tolA* and *tolR* mutants. In another Salmonella study, OMVs from the nontyphoidal Salmonella *tolR* mutant were used to induce the production of IgG antibodies in mice, which reduced bacterial colonization when they were subsequently infected with the wild-type Salmonella strain [89]. Similarly, OMVs from the *S. flexneri tolR* mutant increased CD40 and MHC II molecules and induced the production of the IgG antibodies, and OMVs from the *Shigella boydii tolA* mutant elevated levels of mucosal IgG and IgA antibodies and some pro-inflammatory cytokines, including TNF-α, IL-6 and IFN-γ in mice [42,90]. OMVs from the *H. pylori tolB* and *pal* mutants were shown to induce IL-8 production in human gastric adenocarcinoma cells at a significantly higher level than the wild-type strain [91].

## 4. Pal Proteins for Vaccine Strategies

The bacterial surface molecules, such as lipoproteins, capsular, LPS, pilin, flagellin, serve as antigens to induce protective immunity. In the Tol/Pal system, Pal is a member of the bacterial lipoproteins [23]. Additionally, lipoprotein Pal induces the production of proinflammatory cytokines through the activation of Toll-like receptor 2 (TLR2) [93]. For these reasons, Pal acts as a potential immunogenic antigen to induce both adaptive and innate immune responses.

Some vaccine strategies using the whole recombinant Pal proteins and a synthetic partial peptide have been proposed. Immunization of mice with the recombinant Pal protein from *Legionella pneumoniae* induced the Pal-specific IgG antibodies and a couple of proinflammatory cytokines (IL-6 and TNF-α) and conferred protection against the subsequent *L. pneumoniae* infection [94,95]. Kim et al. determined an epitope of the *L. pneumoniae* Pal protein, responsible for the induction of the immune system, which contains sequences corresponding to amino acid residues 92–100 (Glu-Tyr-Leu-Lys-Thr-His-Pro-Gly-Ala) in the Pal protein [96]. Inoculation of a synthetic peptide containing this epitope sequence together with the CpG-oligodeoxynucleotides (ODN) that is the agonist of TLR9, as an adjuvant, significantly induced cytotoxic T cell responses (represented by elevated IFN-γ and TNF-α levels) and resulted in reduced bacterial burden and protection of mice against *L. pneumoniae* infection. McMahon et al. designed a peptide that contains an epitope sequence, within the *Haemophilus influenzae* Pal protein, to elicit T cell responses [97]. Immunization with this peptide resulted in promoted clearance of the bacteria in mice infected with *H. influenzae*. Spinola et al. established a monoclonal antibody recognizing the Pal protein of *H. ducrevi* from infected patients [78]. Interestingly, this antibody cross-reacted with the Pal proteins from some other Haemophilus species, such as *H. influenzae* [98].

DNA-encoded Pal protein has also been used as the other type of Pal vaccine candidate. A pioneering study demonstrated that the *pal* DNA from *L. pneumoniae* had the ability to induce Pal-specific IgG antibodies and cytotoxic T-lymphocyte responses [99]. In that study, the ability of the plasmid DNA carrying the *pal* gene to induce Pal-specific immune responses was compared with that of the inoculation of recombinant Pal protein into mice for vaccination. Remarkably, mice immunized with the *pal* DNA exhibited stronger cytotoxic T-lymphocyte responses than those immunized with the Pal protein. IgG1 and IgG2a production was also induced after vaccination with the *pal* DNA, though to a moderate extent when compared to that in mice immunized with the Pal protein. One study demonstrated that introduction of the *pal* DNA partially protected mice from *L. pneumoniae* when CD8^+^ T cells are depleted [96]. FlaA and PilE encode flagellin and type IV pilin in *L. pneumoniae*, respectively, and these proteins can act as bacterial surface antigens. Chen et al. used a hybrid DNA construct carrying the *pal* gene together with the *pilE* and *flaA* genes in a single plasmid for vaccination [100]. Vaccination with the plasmid DNA induced some Th1 and Th2 cytokines, including IFN-γ, TNF-α, IL-12, IL-4, and IL-10, together with the IgG antibodies, and protected the mice against the lethal *L. pneumoniae*. The authors also showed that the *pal* DNA vaccine has a higher ability to induce IgGs production compared to the *flaA* and *pilE* DNA vaccines and the vaccine effect of the *pal* DNA can be enhanced by additional administration of *flaA* and *pilE* DNA [100]. In another study, inoculation of DNA encoding the *pal* gene of *Acinetobacter baumannii* induced Th1/Th2/Th17 responses and IgG production [101]. The DNA vaccine conferred protection against acute *A. baumannii* infection in a mouse model of pneumonia, as shown by the alleviated bacterial burden and reduced inflammation in the lungs.

## 5. Conclusions

The Tol/Pal system brings about optimal virulence in many Gram-negative pathogens, while it is also required for bacterial growth and survival in some species. The Tol/Pal system is an effective novel antibacterial target, that is distinguished from conventional targets, such as cell wall synthases, ribosomes, RNA polymerase and primary metabolic enzymes. Therefore, disrupting the Tol/Pal system is a promising strategy in antibacterial therapy. Remarkably, any inhibitors of the Tol/Pal system may be potentially active against refractory multi-drug-resistant (MDR) pathogens that are resistant to several conventional drugs. As one idea, a series of molecules that interfere with a protein–protein interaction or bind any of the TolA, TolB, TolQ, TolR and Pal proteins may be promising compounds to disrupt the Tol/Pal system.

In addition, the Tol/Pal system has considerable immunological properties and can be leveraged for the development of vaccines. The *tol*/*pal* mutants have abilities to elicit protective immunity against the parental wild-type strain and effectively induce an immune response, which may be partly attributed to OMV production in some species. Furthermore, the Pal proteins also stimulate both innate and adaptive immune systems. Pal acts as the ligand of TLR2 to induce proinflammatory cytokines, and also as the antigen recognized by antibodies from infected patients. Thus, the Tol/Pal system may be potentially targeted for vaccine development. Though the *tol*/*pal* mutants were shown to have low virulence, their safety needs to be nevertheless carefully assessed using animal models. The *tol*/*pal* mutants can be generated by inactivating any of the *tolA*, *tolB*, *tolQ*, *tolR* and *pal* genes and these mutants were previously believed to have identical characters. However, the *tolB* and *pal* mutants in *D. dadantii* were shown to be less virulent than the *tolA* and *tolQ* mutants [52]. OMVs from the *tolB* mutant induced IgG production and provided immunity at higher levels compared to the *tolA* and *tolR* mutants [41]. TolB directly interacts with Pal and several other outer membrane proteins, including OmpA required for virulence in some pathogens [18,102,103,104,105]. Therefore, inactivation of TolB or Pal, not TolA, TolQ, and TolR, may affect these outer membrane proteins. We suggest that a comprehensive study of the *tol*/*pal* mutants is essential to characterize which mutant is the lowest in virulence and causes optimal immune responses together with the highest safety.

With regard to vaccine development, a “bacteria-free non-toxin vaccine” based on the Pal protein may be relatively safe compared to a “live-bacterial vaccine” based on the *tol*/*pal* mutants. The *pal* DNA from *L. pneumoniae* induced specific antibodies in mice and its induction was enhanced by administrating together with the *flaA* and *pilE* DNA [100]. Thus, the Pal protein may be served to promote immune responses as another antigen. However, it is noteworthy that excessive induction of proinflammatory cytokines causes severe symptoms. The Pal protein induces proinflammatory cytokines via activation of TLR2; administration of the Pal protein develops bloodstream infection with *E. coli* and accelerates death in mice [36,93]. Therefore, care should be taken to address crucial concerns, such as the optimal dose of Pal-based vaccine to induce protective immunity significantly and to reduce pathological immunity minimally. We expect that Tol/Pal research in the near future would satisfactorily address all these issues.

## Data Availability

Not applicable.
